# Dental Implant and Natural Tooth Micro-Movements during Mastication—In Vivo Study with 3D VIC Method

**DOI:** 10.3390/jpm12101690

**Published:** 2022-10-10

**Authors:** Dániel Tamás Száva, Andrea Száva, János Száva, Botond Gálfi, Sorin Vlase

**Affiliations:** 1 Faculty of Dental Medicine, George Emil Palade University of Medicine, Pharmacy, Science, and Technology of Targu Mures, 540142 Targu Mures, Romania; 2 Department of Mechanical Engineering, Transilvania University of Brasov, 500036 Brasov, Romania; 3 Romanian Academy of Technical Sciences, 030167 Bucharest, Romania

**Keywords:** VIC method, dental implant, tooth, micromovements

## Abstract

In the paper, using the video image correlation method, a study of the micro-movement pattern of the dental implant and of a normal was performed. It is revealed that there are great differences between these two situations. The linear displacement type of the dental implant refers to the linear elastic modulus of bone tissue in the case of normal bite forces. It seems that the major influencing factor regarding the type and value of implant micro-movement is defined by the underlying bone tissue. It is to be considered that masticator force transmission inside a more stiff and dense bone could be attenuated by the antagonist teeth parodontium, dental implant and abutment connection type, and the elastic modulus of material of the dental crown. Because of the elasticity of the periodontal ligament system, during the loading of the dental implant, the natural tooth has been displaced slightly more, leaving the dental implant in an unfavorable position, having to bear the full amount of loading forces. When comparing the relative displacements in the case of the loaded tooth, it is shown that the dental implant has been moving almost symmetrically with the tooth. This could mean that large amounts of forces are transmitted towards the periimplant bone tissue, but in a more optimal, parabolic manner due to the action of the periodontal ligaments surrounding the natural tooth.

## 1. Introduction

Dental implants are widely used in modern dental care, mainly for prosthetic reasons. Their long term durability and applicability is mainly defined by the quality and quantity of the alveolar bone, where they are inserted. For this reason, great efforts are made already from the surgical phases, prior to implant placement for preserving, and in other cases augmenting bone tissue to form a better substrate for the dental implant. Due to the complexity, time consumption, and the high costs of the bone augmentation procedures, the attention has shifted towards the more atraumatic and bone sparing techniques of implant placement and tooth extraction. One of the newest and most innovative and promising techniques is the use of the magnetic mallet [1]. Once the dental implant has been osteointegrated, various types of dental prostheses are connected to their platform. From this moment, the dental implant is exposed to the oral cavity, and loaded with masticatory forces, the alveolar ridge slowly starts to resorb, clearly affecting the long-term success of the dental implant.

Periimplant bone loss after osteointegration is known to be due to biologic and biomechanics factors, which lead to the dental implant failure [2,3].

During mastication, medium to high values of bite forces are transmitted to the alveolar bone through the dental implant superstructure, namely the dental crown. The basic concept of modern implantology assumes that dental implants do osteointegrate, and the dental implant surface gets in direct and intimate relation with the surrounding alveolar bone tissue, without the interposition of any soft tissue. Histological periimplant tissue differs from the normal periodontal ligaments of natural teeth. Natural teeth are anchored inside the alveolar bone, via this pseudo-joint, transforming compressive forces to dragging forces. The ankylotic dental implant instead compresses the bone tissue during masticator loads. Compression of the bone is less tolerated than dragging force, which are much physiological than compressive ones. Besides, periodontal ligament works also as a shock absorber. The biomechanics of the periodontal ligament system was studied and numerically modelled by means of the finite element method (FEM) [4].

Theoretically, loading the dental implant there will be impact like forces transmitted towards the alveolar bone which could initiate bone resorption [5,6]. Other studies conducted on teeth with bone resorption due to parodontal disease have shown that unfavorable loading due to improper occlusion contributed to bone loss [7]. Another study conducted on monkeys showed that periimplant bone loss could only have been observed when the dental implant crown was at least higher than 180 μm compared to surrounding teeth [8].

Supposedly, the periimplant bone tissue has resisted some overloading, even when loads were higher than the loading of the surrounding teeth. This could mean that in some cases, periimplant bone tissue can bear overloading even in case of the missing periodontal ligaments.

The highest compressive forces tolerated without bone resorption vary in case of each bone type, being influenced by the density and the ratio of compact and spongy bone tissue. This is also influenced by age, sex, and constitution. For example, the mandible interforaminal area is much more resistant to bone resorption than any other locations of the maxilla [9,10,11,12,13].

Knowing this, a series of solutions has been discussed to reduce and attenuate some of the loading forces of the dental implant. Dental implant crown morphology has been changed, reducing the occlusal surface. The elastic modulus of the material of the crown has been studied for better stress absorption, and a resilient shock absorber system has been designed for this purpose [14,15,16,17].

For long time, in vivo studies of the biomechanics of the dental implants were considered too laborious and imprecise, and the methods used interfered with the studied phenomena and were consequently abandoned. Regardless, this issue has been often discussed, and solutions for this issue have been implemented in dentistry [18,19].

The video image correlation (VIC) method has successfully been used for in vivo study [20], and the authors also chose this method because of its advantages. Being an optical measurement method, it does not interfere with the studied phenomena.

The aim of this study was to investigate micro-movement types and values in vivo of normal teeth and osteointegrated dental implants, and to collect data regarding the mechanical behavior of the periodontium.

Although in vivo studies are difficult to conduct in the oral environment, the authors consider that they also have many advantages over FEM simulations.

Muscular contraction is known to be the sum of fasciculations of muscle fibres, which are individual, specific, and shape the individual bone quality, and dental occlusion. In this case the loading forces are as real as possible, and are not simplified or distorted [21]. Simultaneously, physiologic dental biomechanics and micro-movements are monitored and can be compared to the dental implant. The masticator forces are transmitted towards the investigated dental implant through the antagonistic natural teeth and are as real as possible. The results of the in vivo investigations show great differences in some cases, due to different measuring techniques, and interferences with the investigated phenomena [22]. The VIC method has no contact with the specimen, and does not interfere with it [19].

## 2. Materials and Methods

To highlight and monitor the movement of the implants and the natural teeth, the authors have used the VIC-3D optical system without contact, from the ISI-SYS GmbH Company, Kassel, Germany, with the software supported by the Correlated Solution Company, Irmo, SC, USA.

In this study, the authors investigated one osteointegrated dental implant positioned on 1.6 (superior maxillary, molar region), the neighboring teeth being the patient’s own and intact teeth, and the masticator forces were performed by means of natural, antagonist, intact, vital teeth.

First, the calibration of the optical system was carried out with the help of a calibration target, a plate provided with a set of black dots arranged at strictly identical distances, with caliber arranged at a distance similar to that of observing the teeth by the optical system VIC-3D. After calibration, the optical system was positioned at the same distance from the monitored tooth.

**Figure 1:**
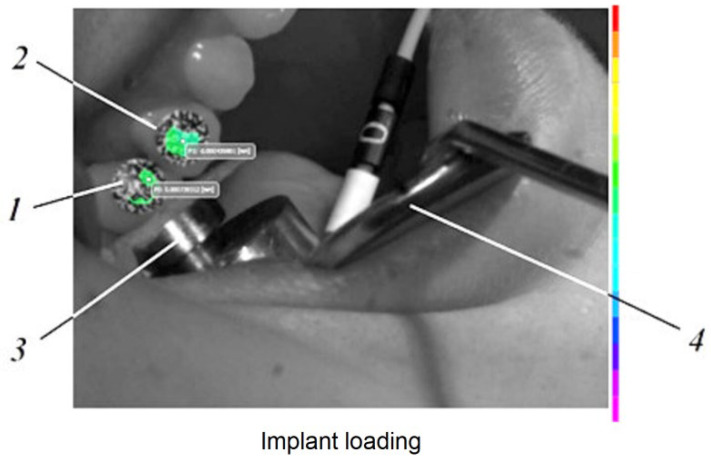
The Implant’s loading capture: *1*—loaded tooth (implant); *2*—unloaded tooth (natural tooth); *3*—load cell; *4*—Farabeuff retractor.

**Figure 2:**
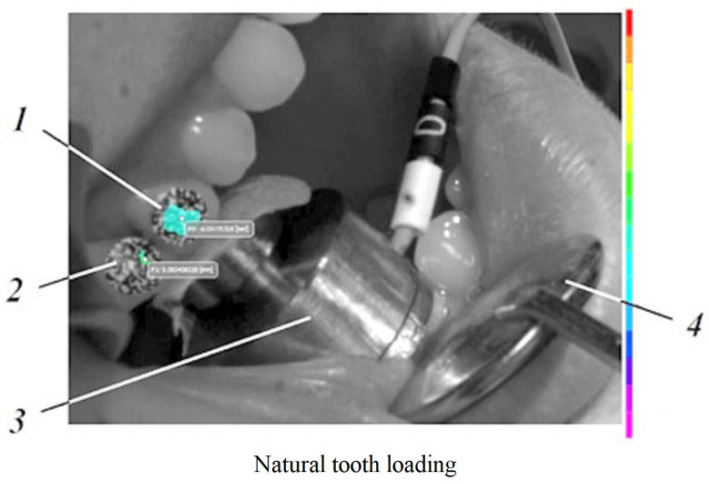
The natural tooth’s loading capture: *1*—loaded tooth (natural tooth); *2*—unloaded tooth (implant); *3*—load cell; *4*—Farabeuff retractor.

On the vestibular surface of the dental crown and the dental implant, which were going to be monitored, sets of black dots with random size and distribution were applied corresponding to the followed area of interest for the selected characteristics of the optical system used for the experiment (zones with the black dots can be seen on Figure 1 and Figure 2).

Along the monitoring of the displacement field, the precise monitoring of the masticator force was also desired. To this end, a miniature (electric-resistive strain gauge) transducer of 1000 N force was used, on which we subsequently applied the actual masticator force.

**Figure 3:**
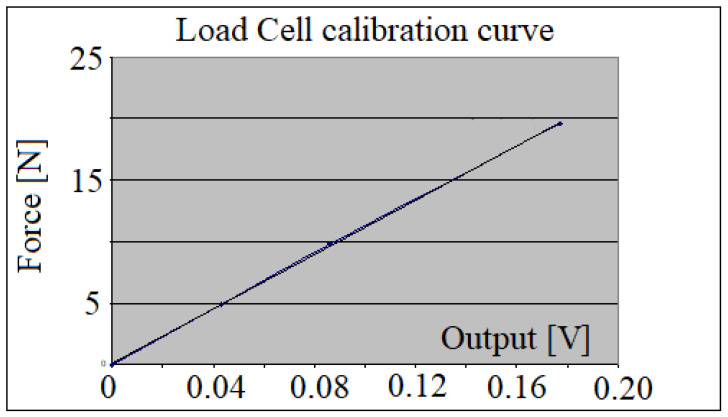
Load cell calibration curve.

Figure 3 shows the calibration curve of the force transducer, where its good linearity can be noted, as well as its sensitivity.

The patient was seated in the dental unit. The force transducer was placed between the dental arches. It will alternately be placed over the surface of the crown of the natural tooth, and the crown of the implant subjected to the tests, situated on the upper jaw, and loaded alternatively.

During the experiments, which consisted of masticator cycles (applying a force and discharging the jaw to a practically unloaded state, ensuring only that the force transducer is held between the teeth), the dentist fixed the patient’s head as still as possible and ensured the retraction of the soft tissues of the oral cavity with the help of a Farabeuff retractor. This was required to ensure the tracking of the area of interest of the oral cavity by the VIC-3D system.

Two sets of measurements were carried out namely:

1. The application of the masticator force on the dental implant and the simultaneous monitoring of the displacements occurring at the level of the implant, and of the mesially situated natural tooth.
2. Applying the masticator force on the natural tooth and simultaneously monitoring its displacements and the displacements of the unloaded dental implant.

Each time, the point marked on loaded crown was marked with P(0), and the point marked on the other (unloaded) crown with P(1).

The displacements of these points P(0) and P(1) marked the primary purpose of these investigations.

In the following figures, a set of similar diagrams are presented in correlation with the rest of masticator cycles where the displacements of points marked P(0) and P (1) appear during the increasing masticator loading forces.

In the case of the loaded implant, both at the implant and at the level of the natural tooth, the characteristics of the curves were practically linear due to the fact that the implant has a practically rigid fixation in the human bone and the corresponding displacement was also according to a linear characteristic. The natural tooth in this case was subjected to a rigid body plane-parallel movement, displacing in a practically similar way to the implant.

In the case of the loaded natural tooth where the fixation in the maxillary bone is elastic, both for it and for the implant, the displacements had a curve corresponding to the elasticity of its fixation area.

Observations: The VIC-3D system has its own reference system, correlated with the position of its two video cameras. During the experimental investigations, the two cameras will capture images related to this system.

**Figure 4:**
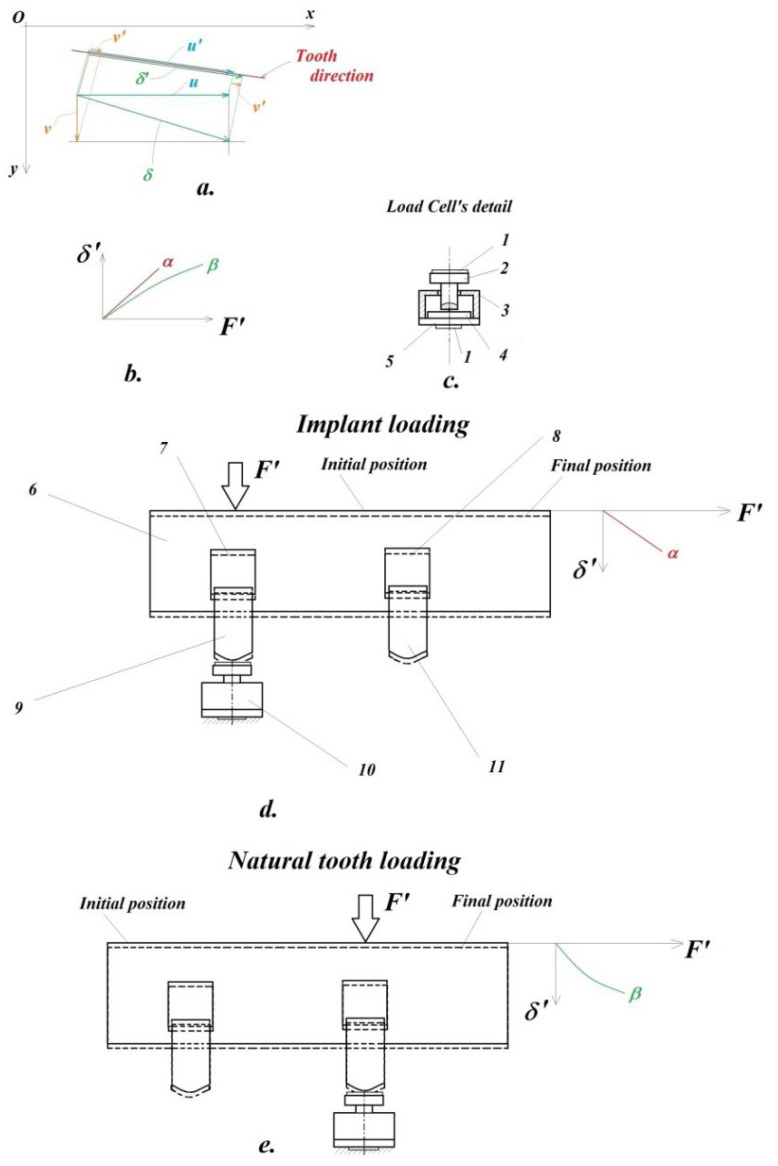
Supplementary details on the analyzed phenomenon: (**a**)—projections’ calculi; (**b**)—linear-, as well as curvilinear characteristic curves; (**c**)—load cell’s detail; (**d**)—plan-parallel movement of the teeth when the implant is loaded; (**e**)—plan-parallel movement of the teeth when the natural tooth is loaded.

The longitudinal axis of the analyzed tooth (marked with the red line; “tooth direction”) forms an angle with the horizontal axis xO of the camera (Figure 4a). In principle, the displacements (u,v) of the point P(0) on the tooth through the resulting displacement vector $\delta =\sqrt{u^{2}+v^{2}}$ (marked with the green line in Figure 4a) must be projected in the direction of the tooth, giving the projection δ’.

Due to the fact that during the simulation of the mastication process both the direction of the applied force *F* (of which only its $F^{\prime }$ projection on the direction of the tooth will be of interest) and the actual direction of the tooth are a little bit changed (there are small involuntary rotations of the patient’s head during mastication), the calculation would become too complicated by the two cameras, a set of components (u,v) would result, for which each time both the resultant δ and its projection δ’ would have to be calculated, completed by monitoring the force projection $F^{\prime }$ in the same direction of the tooth.

In this regard, the authors offer a simpler and more effective solution shown in Figure 4a. Here it can be seen that it will be possible to work/operate much more easily with the projections of the components (u,v) in the direction of the tooth, for i.e., with (u’,v’) the sum of which gives the size of the projection of the resultant δ’, i.e., $\delta^{\prime }=u^{\prime }+v^{\prime }$. This correlation can be demonstrated by a simple mathematical calculation.

In order to monitor the changes in the direction of the applied force, the first approach was to use the average value of the angle formed by the support line of the force (thus the direction of the force) with the average direction of the tooth.

In the case of a rate of image caption of 0.05 s, this stimulated the process of a mastication cycle (cycle of force application and subsequent releasing), lasting for almost a second. In the future, the authors will have to elaborate an automated strategy of data processing regarding the angle arrangement of the tooth and force transmitter.

For suitable analysis of the linear and curvilinear pattern of the displacement diagram (in the direction of the longitudinal axis of the tooth) obtained, in Figure 4, there are offered complete explanations.

If the two crowns (dental implant ***9*** and natural tooth ***11*** are fixed in a medium (mandible) ***6*** having linear force-displacement characteristic (form α from Figure 4b), then according to the principle of parallel plan translation from solid body mechanics (only one crown will be loaded at a time, the other one is left free), both crown displacements will have the same pattern: linear. This corresponds to the loaded implant.

If the medium ***6*** has curvilinear pattern, β (as it is characteristic for the periodontal ligament system of the natural teeth), then both of the crowns will have curvilinear pattern, according to parallel plane displacement.

Figure 4c provides the original solution of the protection of the miniature force transducer ***4***, as follows: The miniature eletrotensometric force transducer ***4*** must be protected from accidental damage during the mastication process, but at the same time the precise, perpendicular orientation of masticatory force must be ensured on the upper center point of this force transducer. Therefore, the authors designed an original support consisting of a steel cylindrical part ***2***, precisely guided (without play, i.e., a sliding adjustment) in the bore of the steel housing ***3***, closed in its lower part with the help of a miniature steel disc ***5***. The transducer of force is also slidably mounted in the cavity of the part ***3***, and the minidisc ***5*** is mounted by gluing with a special adhesive to the housing ***3***. Because during the mastication process, both the crown of the natural tooth and the implant would step directly on the steel surface of the cylindrical part ***2***, respectively, on the minidisc ***5***, a thin layer of protective sheet covering ***1*** of the usual adhesive tape will be interposed. Thus, damage to the teeth that would come into direct contact with the steel surface of parts ***2*** and ***5*** will be avoided.

It Is known from the literature that the natural tooth ***11*** has an elastic fixation, i.e., it forms an elastic medium (similar to the spring-damper subassembly from the suspension of a motor vehicle), by means of the alveolar bone segment (alveolar bone) ***8*** (from Figure 4d), which ensures the mitigation of shocks during mastication, given its force-displacement load curve characteristic (type from Figure 4b). This type of fixation should not be associated/confused with a linear-elastic (force-displacement) characteristic, since the term “elastic” here refers exclusively to the ability of this intermediate layer, i.e., alveolar bone, to absorb shocks during the process of mastication. On the other hand, the currently used implant ***9***, involved in the authors’ investigations, is fixed by means of a double-threaded metallic cylindrical element (sleeve-nut) ***7***, where its outer thread serves to fix it in the mandibular bone 6, and in the inner one fix the pin to the actual implant (implant-bolt). This metal ring ***7*** obviously has a linear-elastic characteristic (type from Figure 4b).

The loading/request each time being applied exclusively on a single tooth, either on the implant, according to Figure 4d, or on the natural one, according to Figure 4e. For an easier understanding of the phenomenon, let’s assume that the applied force, more precisely, its component in the direction of the tooth, i.e., will be taken directly by the requested tooth, and at the level of the miniature force transducer we would have a support point fixed (a simple support from solid mechanics). Due to the stress, the element that will deform will be either the double threaded metallic cylindrical element (sleeve-nut) ***7***, or the alveolar bone portion ***8*** below the natural tooth. Consequently, the connecting element ***6***, i.e., the mandibular bone, will move through a translational movement (from the initial position, marked with a solid line, to the final one, marked with a broken line), carrying the other tooth with it.

Interrupted lines from Figure 4d,e represent the parallel plane displacement of this sub-system of the implant ***9***—natural tooth ***11*** and their connection to mandible ***6***.

In this translation movement, the characteristic of the deformed element ***7*** and ***8*** will be preserved, obviously at a smaller magnitude. Consequently, the unsolicited tooth displacement will exhibit the same characteristic as the above-mentioned deformed element. The mandibular bone ***6***, which connects the two teeth (the implant and the natural one) can be considered in this simplified calculation scheme as perfectly rigid, i.e., non-deformable. However, if the application of the force were to occur somewhere in the interval between the two teeth, then, obviously, both teeth would take shares of this force, and in this case the two involved teeth would present different characteristics from the one mentioned before. Precisely, in order to clearly separate the demand of the two teeth, the authors applied this strategy of individual loading of them (teeth).

The diagrams in the figures showing the displacements experienced by the implant, respectively, the natural tooth, confirm this phenomenon of plane-parallel displacement briefly analyzed earlier.

Moreover, in Figure 1 and Figure 2, those thin layers 1, detailed in Figure 4c, can be observed.

## 3. Results

(1) Case of the dental implant loading with increasing masticator forces.

**Figure 5:**
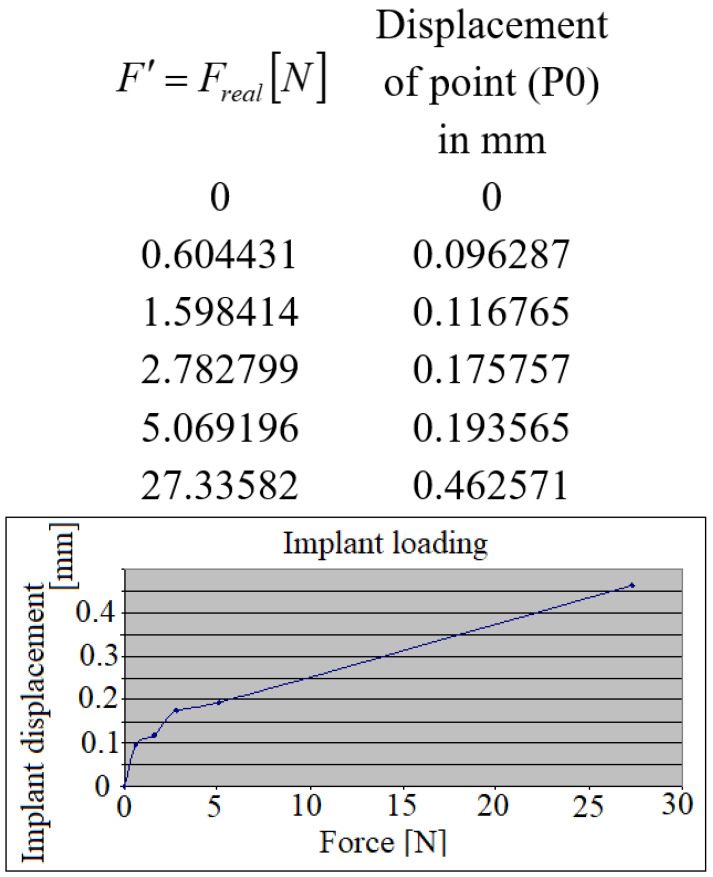
The implant’s displacement during its loading.

Following the evaluation of the collected data during the masticator process, a characteristic cycle of loading (repeated in each of the following cases) could be identified. In Figure 5, the micro-movements of the dental implant crown in its axis, when the implant was loaded (was applied the force F), are presented. Force F[N] from the diagrams represents in fact the projection of the total masticator forces in the direction of the loaded implant crown, i.e., $F^{\prime }=F_{real}\left[N\right]$.

The implant has responded practically in a linear aspect to the masticator forces.

**Figure 6:**
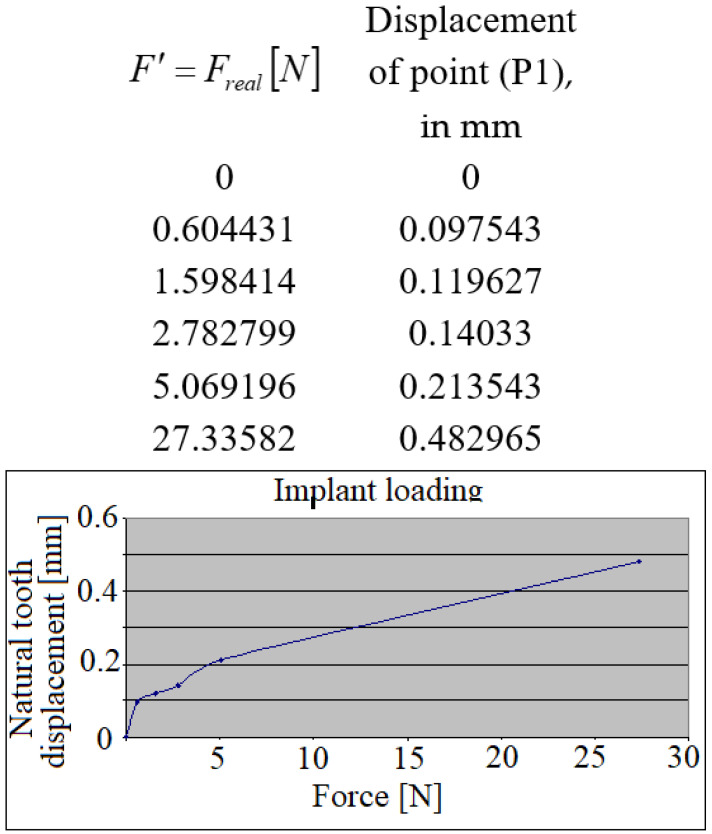
The natural tooth’s displacement during the implant’s loading.

Supplementarily, one has to mention the fact that during the loading of the dental implant, micro-movements of the mesial teeth crown were observed, which as mentioned earlier suffers a parallel plan displacement, clearly remarkable on the diagram in Figure 6.

**Figure 7:**
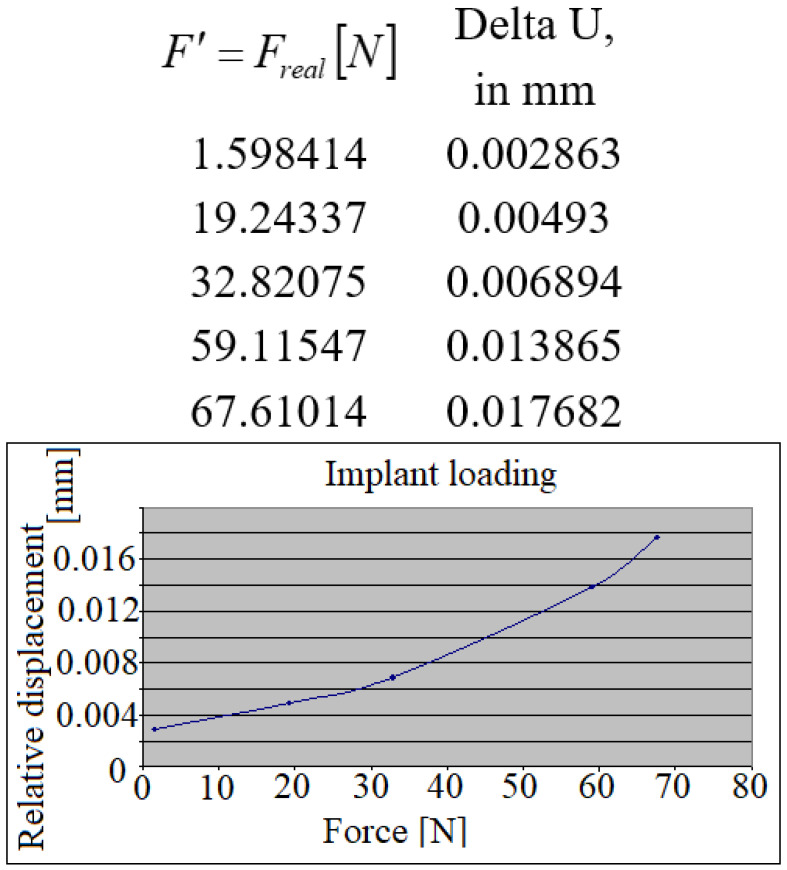
The relative displacements of the crowns during the implant’s loading.

The relative displacements (in absolute values) of the loaded implant/unloaded tooth have also been calculated, as represented in Figure 7. The linear character of the curve can be observed up to 35 *N,* which, subsequently, with the increasing forces changes into a much steeper inclining of the line.

(2) Case of the mesial tooth loading with increasing masticator forces

**Figure 8:**
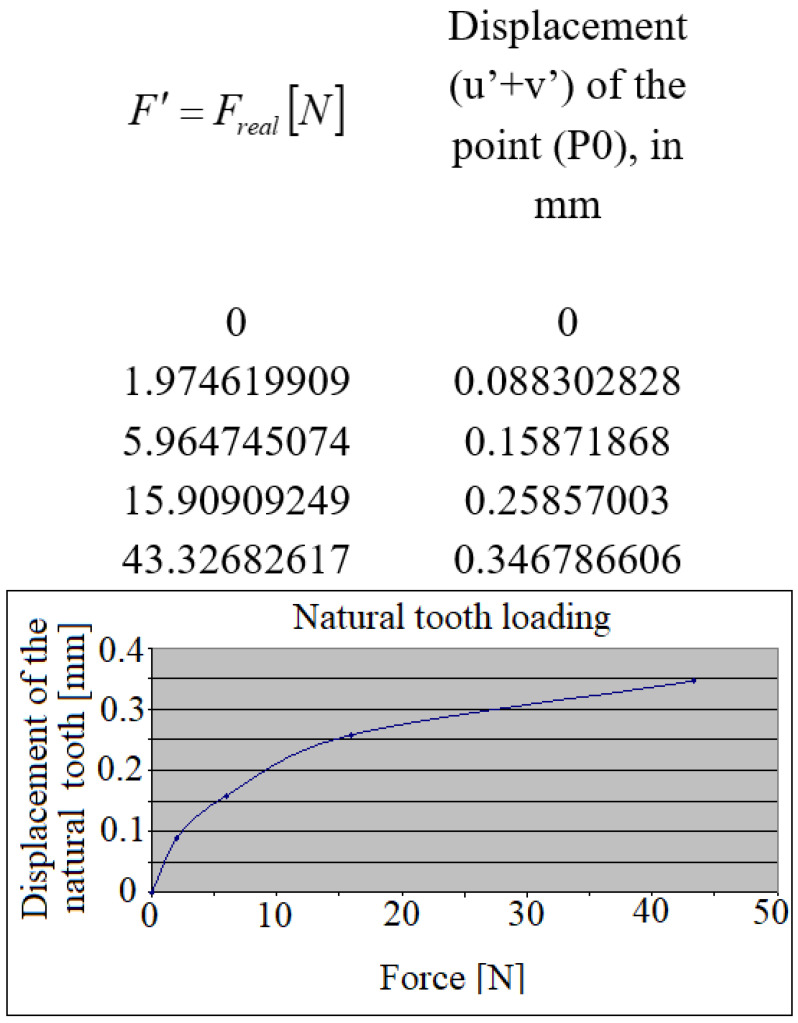
The natural tooth’s displacement during its loading.

Based on similar analysis, a characteristic masticator cycle has been identified, resulting in the diagram shown in Figure 8, representing the displacements in the axis of the loaded natural tooth crown.

**Figure 9:**
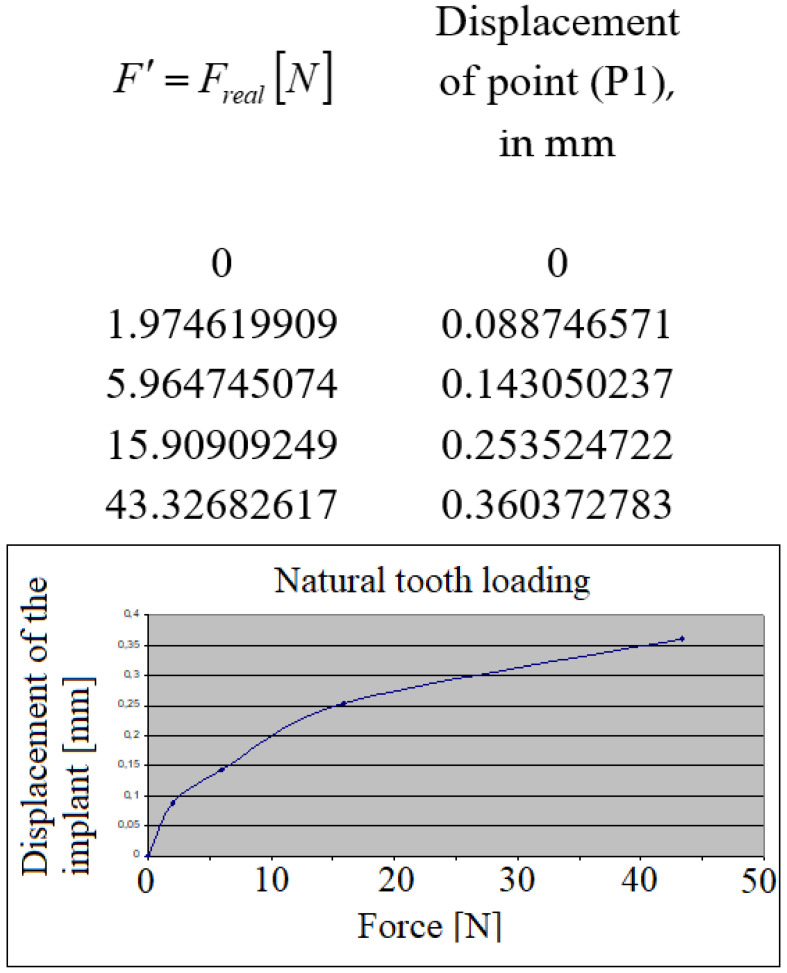
The implant’s displacement during the natural tooth’s loading.

The curvilinear aspect of the response of the periodontal ligament system of the maxillary natural tooth can be observed. Meanwhile, the unloaded dental implant displacements resulted in a similar, curvilinear aspect (Figure 9).

**Figure 10:**
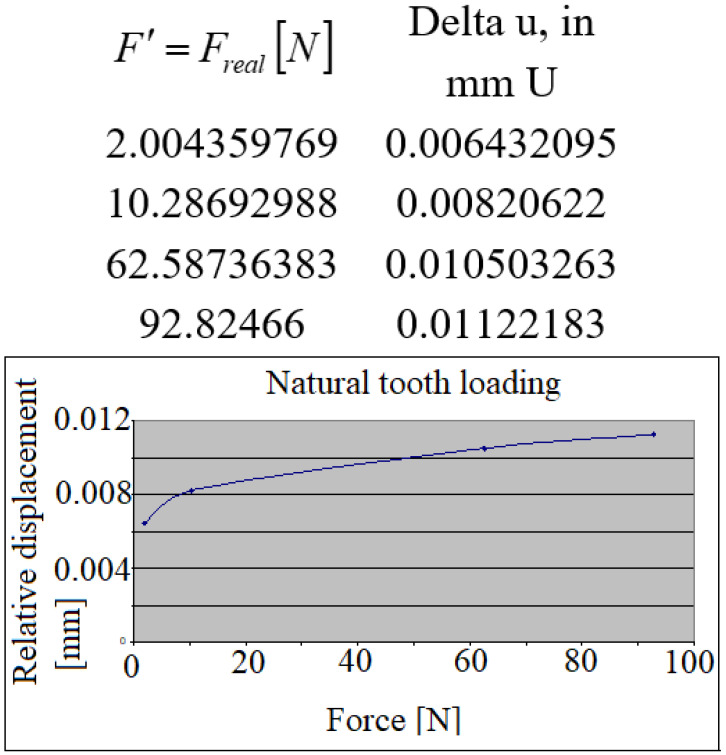
The relative displacement of the crowns during the natural tooth’s loading.

Monitoring the masticator act, in this case, the relative displacements of the unloaded dental implant and the loaded tooth offered the next values, presented in the diagram in Figure 10, where the curvilinear aspect can be observed.

Other important data obtained from this series of experimental investigations concern the correlation of the displacements of the implant and the natural tooth, overlapping their diagrams:

(a) Case of implant loading

**Figure 11:**
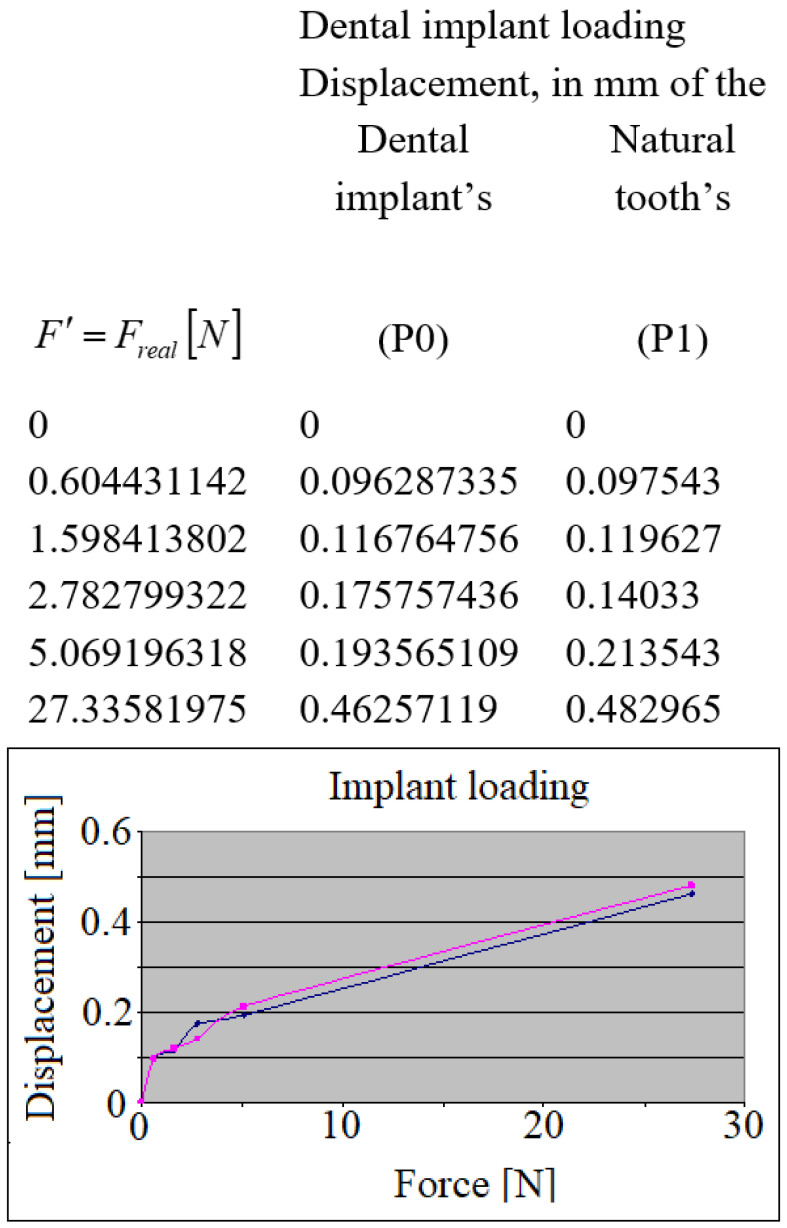
The relative displacement of the crowns during the implant’s loading.

Here, overlapping the analyzed curves, related to the dental implant and natural tooth, resulted in the displacement differences, presented in Figure 11. The linear aspect of the displacements can be observed. The loaded dental implant transmitted linearly the displacements towards the natural tooth through the approximal contact points.

(b) Case of natural tooth loading

**Figure 12:**
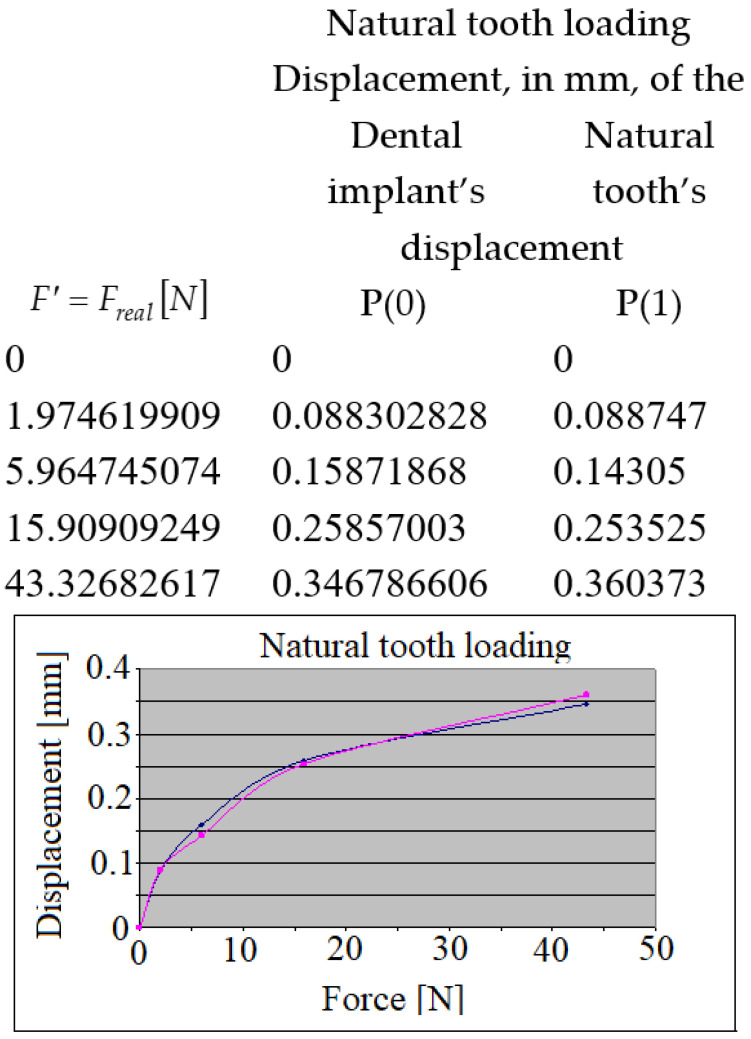
The relative displacements of the crowns during the natural tooth’s loading.

Based on the same procedure, overlapping the curves related to natural tooth loading, the results presented in Figure 12 were obtained. The curvilinear aspect of the relative displacements was observed.

## 4. Discussion

Results clearly show that the micro-movement pattern of the dental implant and the normal tooth show great differences. Approximately similar in vivo experiments produced the same results [5]. The linear displacement type of the dental implant refers to the linear elastic modulus of bone tissue in the case of normal bite forces, up to approximately 50 N [23]. This elastic modulus differs greatly in the case of cortical and spongy bone type. The thickness and proportional distributions of these two bone types define the mechanical properties for each region of the jaws [24]. In our case, absolute values of the micro-movements of the dental implants are defined by the characteristics of this region of the upper jaw, consisting of soft spongy bone, covered by thin cortical bone, reduced much in height by the presence of the maxillary sinus. It seems that the major influencing factor regarding the type and value of implant micro-movement is defined by the underlying bone tissue. It is to be considered that masticator force transmission inside a more stiff and dense bone could be attenuated by the antagonist teeth parodontium, dental implant and abutment connection type, and the elastic modulus of material of the dental crown [25].

In the case of natural tooth loading, where periodontal ligament system was present, the micro-movement pattern type was parabolic, meaning that the loading force transmission has been more optimal than in the case of the dental implant.

Due to approximal contact points between the natural tooth and the crown of the dental implant, it is shown that according to Figure 6 and Figure 9, micro-movements were transmitted towards the unloaded specimen. The micro-movement pattern type present on the unloaded specimen corresponded with the pattern of the loaded specimen.

More valuable are the information presented in Figure 11 and Figure 12, which represent the relative micro-movements of the dental implant and the natural tooth. Because of the elasticity of periodontal ligament system, during the loading of the dental implant, the natural tooth has been displaced slightly more, leaving the dental implant in an unfavorable position, having to bear the full quantity of loading forces. When comparing the relative displacements in case of the loaded tooth, it is shown that the dental implant has been moving almost symmetrically with the tooth. This could mean that great amounts of forces are transmitted towards the periimplant bone tissue, but in a more optimal, parabolic manner, due to the action of the periodontal ligaments, surrounding the natural tooth.

**Figure 13:**
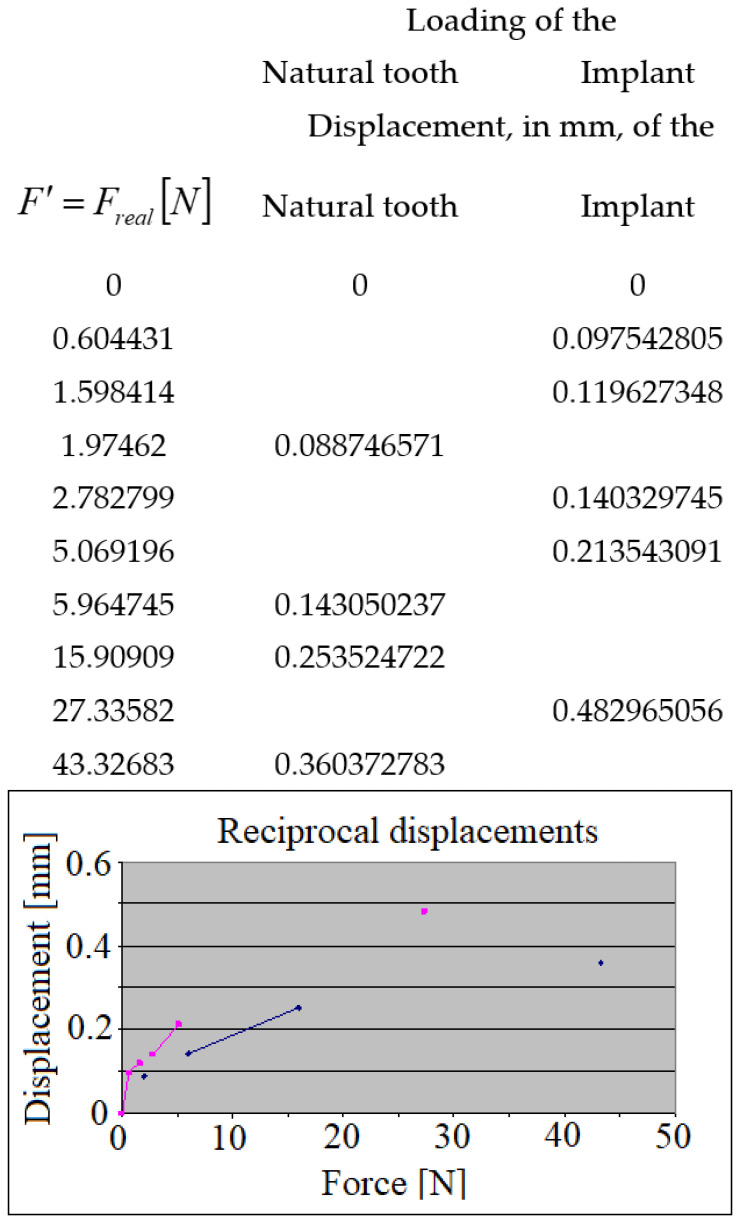
The reciprocal displacements of the crowns during the mastication: the implant’s displacement during the natural tooth’s loading (blue), as well as the natural tooth’s displacement during the implant’s loading (red).

When comparing the reciprocal displacements (Figure 13), the implant’s displacement during the natural tooth’s loading (blue points/lines), and the natural tooth’s displacement during the implant’s loading (red points/lines), it is found that the natural tooth presents higher mobility than the dental implant, being capable of bearing higher loads than a dental implant.

Another useful and important aspect will be to elucidate the method of transmission of force applied by several teeth, such as: two adjacent natural teeth, one implant and one natural tooth, as well as two adjacent implants, but also other useful cases, found in dental practice.

Obviously, this study represents only the beginning of complex investigations that the authors intend to carry out in the coming period.

Even if it seems a relatively simple strategy at first glance, the practical completion of each new type of test requires a different approach.

## 5. Conclusions

The viscous-elastic properties of the bone increase its resistance on loading forces that act for short periods of time [26]. Bone can adapt better in the case of cyclic loadings than long-acting, static ones, which provoke bone resorption. Bone regeneration requires resting periods, with no mechanical loading period [27]. One masticator cycle takes approximately 0.8–1 sec, short enough for the bone tissue to act like a viscous-elastic medium, although it has not modified the linear displacement type of the dental implant. In this case, at least for this softer bone type, the viscous-elastic characteristics of the bone could not be shown and the attenuation of stresses was present. The linear micro-movement pattern in the case of the dental implant was present.

The authors tried a new approach (to the best of the authors’ knowledge not currently used) of the mastication process in vivo with the help of a high-precision optical system and without direct contact with the studied area. The results obtained are promising, even if this methodology can be improved based on further investigations. This original approach will certainly open new ways toward deepening the understanding of the mechanism of mastication, bringing significant information that could not be obtained based on existing in vitro investigations by specialists.

In the future, the authors would like to continue more detailed investigations regarding dental implants inserted in different areas of the maxilla and mandible characterized by different bone quality. Multiple and splinted dental implant biomechanical behavior investigations will also be performed in the future.

## Data Availability

Not applicable.
